# Correction of Post-Surgical Temporal Hollowing with Adipo-Dermal Grafts: A Case Series

**DOI:** 10.3390/jcm13164936

**Published:** 2024-08-21

**Authors:** Stefano Andreoli, Davide Brucato, Daniel Schmauss, Yves Harder

**Affiliations:** 1 Department of Plastic, Reconstructive and Aesthetic Surgery, Ospedale Regionale di Lugano, Ente Ospedaliero Cantonale (EOC), 6900 Lugano, Switzerland; stefano.andreoli@hotmail.com (S.A.); davidebrucato1@gmail.com (D.B.); daniel.schmauss@eoc.ch (D.S.); 2 Faculty of Biomedical Sciences, Università della Svizzera Italiana, 6900 Lugano, Switzerland; 3 Faculty of Medicine, Technical University Munich, 81675 Munich, Germany

**Keywords:** reconstructive surgery, adipo-dermal graft, fat grafting, temporal hollowing, neurosurgery

## Abstract

**Background:** Surgical dissection and partial detachment of the temporalis muscle during neurosurgical procedures might result in a temporal depression known as “temporal hollowing”. Reconstructive procedures to correct this condition include the implantation of synthetic materials (e.g., hydroxyapatite, methacrylate or polyethylene), injection of autologous fat or fillers as well as the use of flaps (e.g., temporo-parietal local flap and latissimus dorsi free flap). However, none of these techniques proved to be an ideal option due to a high complication rate or need for invasive and complex surgery. Adipo-dermal grafts are autologous; the resorption rate seems to be limited and the complexity of the procedure is minor. Moreover, autologous fat grafting can be performed to enhance the final outcome by correcting residual contour deformities. **Methods**: In this series of five patients, an institutional experience is presented using multi-layered adipo-dermal grafts harvested from the supra-pubic area for the restoration of volume and contour in post-surgical temporal hollowing. During the last follow-up, patients were asked to express their satisfaction, which was assessed by a survey. **Results**: this approach demonstrates a marked improvement in temporal hollowing associated with a low complication rate and good patient satisfaction. **Conclusions**: the aim of this consecutive case series is to emphasize the effectiveness of this surgical technique as one of the options to address temporal hollowing.

## 1. Introduction

Soft tissue depression in the temporal area may occur after neurosurgical dissection of the temporalis muscle, which is a condition known as “temporal hollowing”. It may result from rather standardized surgical neurosurgical approaches, including fronto-temporal or coronal incision, including a more or less extensive detachment of the temporalis muscle from its underlying bone. This may cause a retraction and subsequent atrophy and scarring of the muscle itself. This volume defect is reported to occur after 87 to 100% of craniectomies or craniotomies in the temporal region and may result in a patient’s lack of confidence and psychosocial discomfort [1,2]. Moreover, in some patients this discomfort is represented not only by the volume defect per se but also by the caudally adjacent tissue bulge resulting from the retracted temporalis muscle. Several surgical techniques have been proposed to prevent temporal hollowing, one of which suggests avoiding detachment of the deep temporal fascia or intercalated temporal fat pad [3]. Another technique involves the use of a split myofascial bone flap for craniotomy [4].

If not preventable, several reconstructive approaches have been described to correct temporal hollowing. However, these procedures differ significantly in invasiveness and complexity. Currently, there is no so-called “gold standard” but rather a case-by-case approach. 

One option is the implantation of “customized” synthetic materials, such as hydroxyapatite [5], methyl methacrylate [6] or porous high-density polyethylene [7]. Although long-term results have been reported to be satisfactory, the foreign body response, rather high rate of encapsulation and infection and risk of extrusion limit their use [8]. 

For facial reconstruction in general, the use of local and regional flaps, including a temporo-parietal fascial flap, pedicled trapezius flap, pectoralis major flap or latissimus dorsi flap have been described, but none of them has proven to be suitable to address temporal hollowing [9].

The use of microvascular flaps has shown consistent results in the literature, but their use should be limited to selected cases due to the need for rather complex surgery resulting in potentially relevant “collateral damage” at the donor site following flap elevation [10]. 

Injections of fillers or autologous fat harvested by means of liposuction are known alternatives to correct temporal hollowing. These approaches are technically easier and less invasive and can be performed in an outpatient setting. However, in addition to the unpredictable reabsorption rate of fillers or autologous fat, particularly when injected in an already adherent and scarred tissue [11], severe or permanent complications such as vision loss or skin necrosis have been described after facial augmentation in this area [12,13]. Given these drawbacks, none of these techniques have become the standard for treatment to correct temporal hollowing.

As a matter of fact, adipo-dermal grafts represent a well-described technique for soft tissue augmentation [1,14]. This procedure is well-established and has been used to reconstruct craniofacial defects and to augment soft tissues of the face for aesthetic purposes [14,15].

The aim of this case series has been to evaluate our institutional experience using adipo-dermal grafts for the reconstruction of post-surgical soft-tissue defects in the temporal region. 

## 2. Surgical Technique and Patient Evaluation

All patients that have been included in this case series were referred from neurosurgeons to our clinic due to complaints about temporal hollowing. Thereafter, the below-mentioned surgery was offered as a therapeutic option. All patients were otherwise healthy and consented to the use of clinical data upon signing an informed consent.

After assessing pre-existing surgical scars, concavity in the temporal area, including quality of the skin and underlying muscular tissues, and residual muscular function were evaluated. Evaluation of the absolute depth of the defect and caudal bulging of the retracted temporal muscle was key to define the expected extent of the deformity at depth, horizontally and vertically.

Thereafter, a donor site for skin was identified. Since these patients required a multi-layer skin graft, the lower abdomen in the supra-pubic area was shown to be an adequate donor site, resulting in a rather inconspicuous scar. Preoperative markings were performed while the patient was sitting, outlining the graft to be harvested and marking the temporal depression as well as the caudal bulge of the muscle (Figure 1A). 

Surgery was usually performed in an outpatient setting, under general anesthesia in a standard operating room and in a supine position, providing free access to both the temporal region and the abdomen. Preparation of the recipient site and harvesting of the graft was then performed in a “two-team” approach, when possible.

A skin incision was made as needed using the pre-existing scar to access and prepare the recipient site according to the preoperative markings and the defect’s extension. This included detachment of the skin and subcutaneous tissues from the deeper planes, usually consisting of periosteum cranially, and scarred and retracted temporal muscle caudally. Scar release and excision of fibrotic tissue was performed as needed, as well as surgical debulking of the retracted muscle, in order to be able to minimize residual peripheral bulging in the peri-zygomatic region.

Thereafter, the extent of the footprint of the defect (which was based on the longest horizontal and vertical diameter) and the depth of the cone-shaped defect were measured with a sterile ruler. Using a paper template, the total size of the graft was then defined and the supra-pubic skin was marked accordingly and incised (Figure 1B). The graft to be excised was first de-epithelialized and then harvested as a “dermal graft” with a thin underlying layer of subcutaneous fat, which was subsequently completely defatted if needed. 

According to the defect’s depth, the thickness of the graft to be inserted can be increased using an overlay of multiple graft layers to best reconstruct the depth of the defect and possibly its volume. Since graft thickness after de-epithelialization may drastically vary from patient to patient, it is almost impossible to decide preoperatively upon the total number of graft layers to use. It is worth considering that defects usually have a concave and conical shape. In order to “prefabricate” the multi-layered graft, individual graft layers were extended and sutured one upon the other using an absorbable 5-0 suture (i.e., polyglactine: Vicryl Rapide), avoiding retraction as much as possible, and 2–3 grafts were superimposed to provide the thickness needed (Figure 1C). The aim was to slightly overcorrect the depression, given the partial reabsorption of fat, if present, and retraction of the dermal graft. The “customized” graft was then pulled through the prepared pocket using 8–10 non-absorbable transcutaneous “pulley” stitches (i.e., polyamide: Ethilon 3.0) that were secured to the skin over a dabber or similar (Figure 1D,E). The surgical incision was closed after hemostasis and rinsing with saline, usually using a layered closure and absorbable sutures both at the recipient and donor sites. A surgical drain is not always necessary since slight compression is applied over the recipient site. Patients received 5 days of post-op antibiotic prophylaxis (Cefuroxime, 1 g/24 h). The transdermal stitches were removed after a minimum of 3 weeks to ensure that the grafts were already adhered to the surrounding overlaying skin and periosteum at depth. 

Body Mass Index (BMI) was calculated before surgery and at 3 months after surgery to evaluate if a change in BMI may influence the graft volume. 

Every planned outpatient visit before and after surgery included not only patient history and a clinical evaluation but also standardized photo documentation to ensure an objective and comparable evaluation of the hollowing and its correction over time. The views included frontal, 45° as well as 90° left- and right-side views using a standardized light and background. 

A follow-up of 12 months has been defined as the minimal one, assuming that graft integration and potential shrinkage and or displacement would have occurred by this time period. On the occasion of the last follow-up, patients were asked to self-evaluate their satisfaction using a standardized, though not validated, questionnaire, including the final result, changes in self-confidence and general satisfaction about the treatment received. The questionnaire included a four-point scale with “1” representing a poor result, “2” a sufficient result, “3” a good result and “4” an excellent result. This questionnaire allowed for reproducible and quantifiable assessment of patient satisfaction, providing valuable feedback on the effectiveness of the treatment.

## 3. Patient’s Presentation

### 3.1. Patient Number 1

A 43-year-old man who underwent an emergency decompressive craniectomy using a fronto-temporal access on the left to treat an ischemic stroke of the middle and anterior cerebral artery. One year later, the patient presented a facial contour deformity on the left with extensive temporal hollowing and a peri-zygomatic bulge (Figure 2A). The temporal depression was reconstructed using a double-layered adipo-dermal graft harvested from the supra-pubic area. At 3 months, the patient was moderately satisfied due to residual temporal hollowing (Figure 2B). As a consequence, the patient underwent a second surgery, using another double-layered adipo-dermal graft and additional surgical debulking of the retracted temporal muscle at the zygomatic arch. At 12 months, temporal hollowing had significantly improved (Figure 2C).

### 3.2. Patient Number 2

A 64-year-old woman who underwent an elective pterional craniotomy to remove a meningioma in the sphenoid region on the right. One year later, the patient was concerned about the surgical scar and significant temporal hollowing (Figure 3A). Contour deformity was first addressed using a triple-layered adipo-dermal graft harvested from the supra-pubic area. Three months later, temporal hollowing was still visible (Figure 3B). Therefore, 30 mL of autologous fat harvested from the periumbilical region using a modified Klein’s solution was infiltrated locally. Following initial ingrowth and a satisfactory take rate of the fat, the patient returned to our department due to some loss of volume of the grafted autologous fat that was associated with weight loss of the patient (Figure 3C). Therefore, a second triple-layered adipo-dermal graft was used to correct contour deformity. Twelve months later, the patient reported a visible improvement in the temporal area with significantly less visible temporal hollowing (Figure 3D).

### 3.3. Patient Number 3

A 23-year-old man who underwent an emergency unilateral fronto-temporal decompressive craniectomy on the right following the rupture of a previously unknown arterio-venous malformation. Two years later, the patient complained about significant facial asymmetry resulting from temporal hollowing associated with severe peri-zygomatic bulging (Figure S1A). Two sessions of scar release and grafting of 20 mL and 30 mL of autologous fat, respectively, harvested from the lower abdomen and the flanks resulted in a minor improvement in the patient’s satisfaction (Figure S1B). Thereafter, a double-layered adipo-dermal graft was inserted after harvesting it from the supra-pubic area. Eight days after the intervention, early removal of the stiches caused a slight displacement of the graft, which was probably due to insufficient adherence to the adjacent tissues. Nevertheless, 18 months later, temporal hollowing and the peri-zygomatic bulge were markedly improved (Figure S1C).

### 3.4. Patient Number 4

A 44-year-old man who underwent an emergency bilateral fronto-temporal craniectomy to treat a complex skull fracture. At the one-year follow-up, severe bilateral and asymmetric temporal hollowing associated with peri-zygomatic bulging was observed (Figure 4A). A bilateral double-layered adipo-dermal graft harvested from the supra-pubic area was performed. One week after surgery, the patient developed an infected seroma, which was completely resolved with drainage in the outpatient clinic and oral antibiotic therapy without any further surgical revision. Visible improvement was already observed at the 3-month follow-up (Figure 4B). Nine months later and due to soft and non-adherent tissue conditions, bilateral scar release and grafting of 40 mL of autologous fat per side were performed. A second session of autologous fat grafting of 30 mL on the left side and 20 mL on the right side was performed after another six months, resulting in an additional improvement in contour deformity at 12-month follow-up (Figure 4C). 

### 3.5. Patient Number 5

A 34-year-old man who underwent an emergency decompressive bi-coronal craniectomy following a severe cranio-cerebral injury. One year later, bilateral temporal hollowing and peri-zygomatic bulging were observed, significantly disturbing the patient (Figure S2A). After harvesting skin in the supra-pubic area, a double-layered adipo-dermal graft was inserted bilaterally with improvement of the contour deformity at 12-month follow-up (Figure S2B). Seven months later, the patient underwent bilateral scar release with grafting of 60 mL of autologous fat on the right side and 30 mL on the left side. 

## 4. Results

The patients’ demographics and surgical history are listed in Table 1. All procedures were performed between 2020 and 2023. The mean age of the patients was 41.6 years. The mean BMI moderately increased before and after surgery (mean BMI before surgery: 24.6; after surgery: 26.6). In fact, four out of five patients gained weight, except for patient number 2 who lost weight following fat grafting, impacting on graft take and eventually volume restoration. Three patients underwent unilateral correction of the post-surgical temporal deformity, whereas two patients underwent bilateral correction. In all cases, the supra-pubic area served as the donor site with all of them presenting with uneventful healing and eventually scarring. The number of layers per “customized” graft ranged from two to three with a mean of 2.3 layers per graft. The mean follow-up time after the last surgery was 22 months (range: 13 to 27 months). Three out of five patients underwent additional autologous fat grafting procedures in order to correct residual contour deformities (Patients 2, 4 and 5). Patient 3, on the contrary, underwent autologous fat grafting as a primary procedure without showing any effect and, therefore, decided to correct the deformity using adipo-dermal grafts.

The postoperative result was graded as good in three cases and sufficient in two patients, resulting in a mean outcome of 2.6 (range 1–4). 

## 5. Discussion

Adipo-dermal grafts were first described by Figi et al. in 1931 to improve post-traumatic contour deformities of the face [16]. In 2012, McNichols et al. described the use of adipo-dermal grafts to correct temporal hollowing in a personal case series of five patients [1]. The authors used a single-layered adipo-dermal graft harvested from the abdominal region. However, neither scar release nor extensive surgical resection of the surrounding fibrotic tissues and retracted temporal muscle creating the bulge were described. Nevertheless, according to the authors, the use of this type of graft was demonstrated to be a valid option to treat this condition with improvement of the contour defect. 

Compared to synthetic materials, such hydroxyapatite [5], methyl methacrylate or high-density porous polyethylene [6,7], autologous tissue in general and adipo-dermal grafts in particular seem to represent better options due to their biocompatibility allowing ingrowth into the surrounding tissues and guaranteeing a certain suppleness over time and resistance to infections [15]. According to McNichols et al. and Kumar et al., adipo-dermal tissue can be harvested easily; the grafts can be tailored “ex situ” to best fit the defect; and surgical morbidity is low [1,15].

This consecutive series describes our experience of nine consecutive surgeries using adipo-dermal grafts to treat a total of seven contour deformities in five patients. Overall, the procedure showed a low complication rate, as also stated by other authors [15,17]. In one case, early removal of the “pulley” stitches resulted in partial retraction of the graft, suggesting that secondary graft displacement may occur, particularly if ingrowth of the graft with the surrounding tissues has not yet happened, particularly if residual seroma is still present during the first days after surgery. Accordingly, we believe that stitches need to remain for a minimum of 3 weeks, followed by individual “case-to-case” decisions regarding the correct time for stitch removal. Moreover, although autologous, multi-layered adipo-dermal grafts might be more prone to infection, as it has been observed in one patient in this series. Its thickness may be associated with delayed engraftment and eventually incomplete integration, particularly of the central layers or core region of this three-dimensional graft when compared to a single-layer graft. Other potential complications of this procedure are above all related to surgery and include hematoma, complete graft reabsorption or development of pathological scars both at the donor and recipient sites. To best lower the rate of complications, surgery has to be executed in a meticulous way. Nevertheless, these types of complications have not been observed, neither in our cases nor in the literature.

One potential drawback of this procedure when compared with the use of synthetic fillers might be the need for a donor site, which is associated with potential “collateral damage”, such as bleeding, infection, wound dehiscence or pathological scarring. However, so far, neither the current literature nor the herein presented cases describe any complications related to the donor site. It is worth mentioning that none of the operated patients presented risk factors such as smoking, diabetes mellitus or chronic vascular obstructive disease.

Autologous fat grafting has proven to be a valid alternative to correct adherent scars [18,19,20] and post-surgical or post-radiotherapy contour deformities and to restore skin quality in ageing patients [21,22,23]. For the reconstruction of severe temporal hollowing as presented here, autologous fat grafting has shown to be associated with some limitations if used as the “first line” treatment. 

For example, it does not seem to be a suitable and durable approach in cases of rather thin skin and scar tissues adherent to the periosteum of the temporal bone. As a matter of fact, the absence of a real three-dimensional space does not enable adequate injection of fat in these tissues and improves tissue quality rather than increases tissue thickness. 

Furthermore, Sheri et al. demonstrated that adipose tissue from high-BMI donors might have a greater graft take rate. Inversely, the authors postulated that a low to normal BMI might hamper the take rate of injected autologous fat [24]. In this regard, it has to be mentioned that the majority of the herein presented patients presented with a mean BMI of 25 kg/m^2^. 

Moreover, as reported by various authors focusing on different regions of the body, it has been shown that the resorption rate of fat depends on many factors and is rather unpredictable, ranging from 20 to 90% [25]. In the facial area, for example, resorption of grafted autologous fat has been reported to occur up to 47% [18], as demonstrated by Lv Q et al. in a cohort of 1011 patients undergoing injection of autologous fat into the face for reconstructive purposes. It is also known that changes in body weight could influence the volume of grafted fat, as has been experienced by one patient who significantly lost weight after autologous fat grafting, presumably impacting on the grafted adipose tissue.

Choi J et al. reported satisfactory outcomes using autologous fat grafting to treat temporal hollowing resulting from diverse conditions [11]. In this series, however, as experienced in one of our patients, autologous fat grafting alone has not been an option to definitively correct contour deformity in the temporal area. We, however, believe that autologous fat grafting is effective to improve the quality of the recipient site’s tissues, resulting in softer tissues, which more easily allow subsequent correction of residual contour irregularities after inset of the graft rather than an increase in tissue thickness.

In fact, it is well-known that the take rate of injected autologous fat is greater with the introduction of a scaffold, which can be liquid or solid, autologous or synthetic [24,26]. Natural scaffolds, such as poly(lactic-co-glycolic) acid or polyethylene glycol, are considered the most suitable ones for graft take [24,26]. Accordingly, we believe that adipo-dermal grafts may possibly act as an “autologous scaffold” of soft and well-vascularized tissue for subsequent injection of autologous fat to increase its volume.

Papel et al. demonstrated that adipo-dermal grafts as a “stand-alone” procedure offer improved predictability and longevity of the outcome in reconstructive surgery of the face when compared to the use of autologous fat grafting alone [25,27]. We, therefore, believe that a combination of the two techniques, i.e., surgical debridement and inset of customized adipo-dermal grafts followed by autologous fat grafting as needed is the way to treat temporal hollowing. 

In cases of persistent hollowing due to insufficient volume correction as well as retraction or inadequate shrinkage of the adipo-dermal graft, a “redo” of the procedure must be taken into consideration, particularly if the initial hollowing is severe. Despite a redo rate of two out of five patients, patient satisfaction was good. 

Moreover, successful correction of these significant contour deformities not only depends on graft take but also on how the present bulge of retracted muscle at the zygomatic arch can be reduced. In our experience, any resuspension or reattachment of this retracted and scarred muscle is barely possible. In addition, reduction of the bulge is challenging due to the presence of the temporal and zygomatic branches of the facial nerve, which might be injured during the debulking procedure. 

Postoperative clinical and photographic follow-up and patient satisfaction using standardized photographs and a standardized questionnaire, respectively, show encouraging results. Descriptive case series present some limitations that could influence the interpretation of the findings. Firstly, the absence of a matched untreated control group somehow limits the causal correlation about the effectiveness of the adipo-dermal graft and its outcome. In three out of five patients, autologous fat grafting has been used as an additional procedure to correct residual contour deformities after the inset of adipo-dermal grafts. Although the outcome does not correlate with the type of procedure (adipo-dermal grafts in combination with autologous fat grafting do not necessarily yield better results than adipo-dermal grafts alone), it may be assumed that the sequential use of adipo-dermal grafts followed by autologous fat grafting as needed may improve the final outcome. Nevertheless, all presented patients decided to undergo surgery because they were unhappy with the condition of more or less advanced post-surgical temporal hollowing. Interestingly, following surgery, they rated their outcome as sufficient in two cases and good in three cases. Secondly, patient history, clinical evaluation and photo-documentation are limited sources for objective outcome measurements to be quantified. Thirdly, the small sample size evaluation of patient satisfaction is based on some subjective criteria.

## 6. Conclusions

This consecutive case series demonstrates that multi-layered adipo-dermal grafts are a reliable option to restore post-surgical temporal hollowing and are associated with good patient satisfaction. The surgery is rather easy to perform, usually in an outpatient setting using a standard operative theatre, and the complication rate is low. Inset of the graft in combination with excision of the scar tissue may provide a well-vascularized and soft bed for subsequent autologous fat grafting to correct residual deformities if needed.

## Figures and Tables

**Figure 1:**
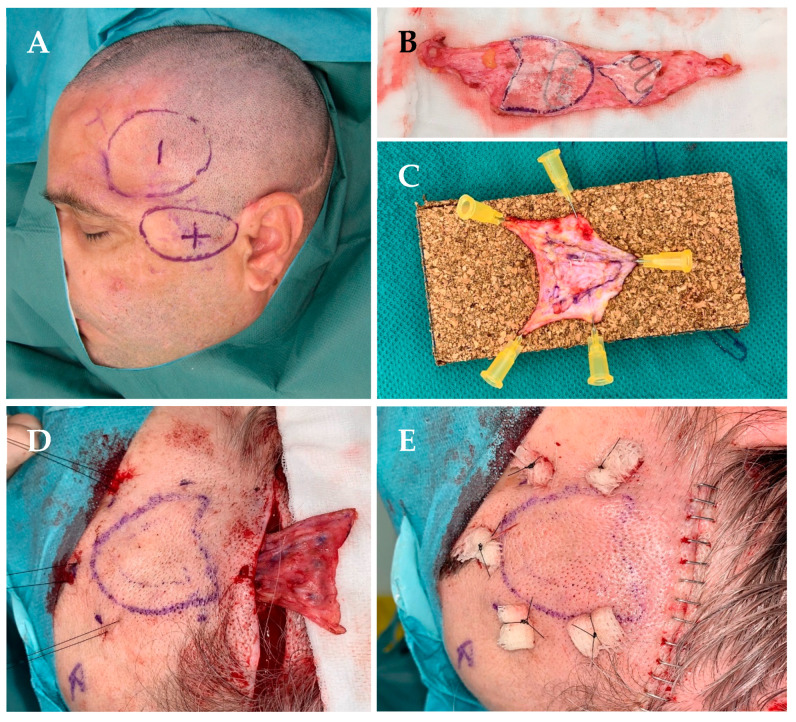
(**A**) Preoperative markings of the temporal area showing the depression (“temporal hollowing” marked as −) and peri-zygomatic bulge (marked as +). (**B**) Adipo-dermal graft harvested from the supra-pubic area after de-epithelialization with two paper templates in place before tailoring and suturing the customized graft. (**C**) Double-layered adipo-dermal graft showing the larger graft on the bottom, fixed on a corkboard. (**D**) Pull-through of the “prefabricated” graft into the pocket after having placed some “pulley” sutures. (**E**) After fixing the graft with transcutaneous “pulley” sutures and a layered closure of the surgical access.

**Figure 2:**
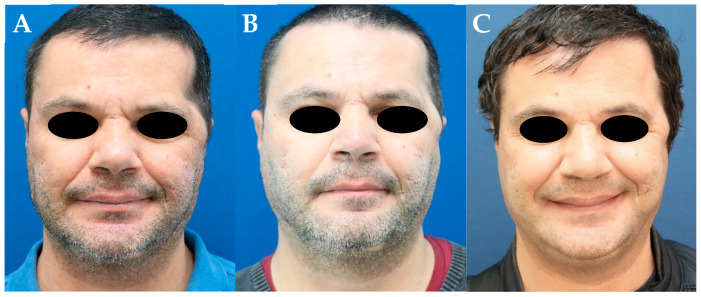
(**A**) Significant temporal hollowing and a peri-zygomatic bulge on the left after a fronto-temporal craniectomy. (**B**) Three months after the inset of a double-layered adipo-dermal graft harvested from the supra-pubic area. (**C**) Twelve months after a second stage using another double-layered adipo-dermal graft, which was again harvested from the supra-pubic area.

**Figure 3:**
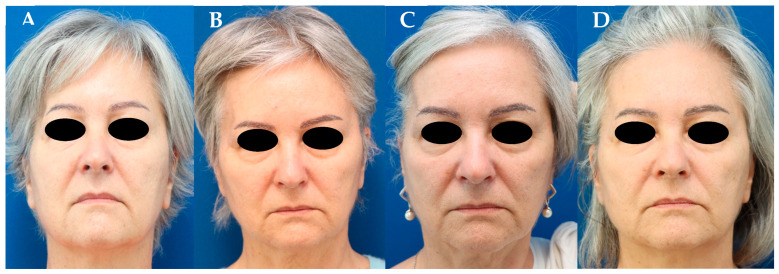
(**A**) Visible temporal hollowing on the right after a pterional craniectomy. (**B**) Three months after the inset of a triple-layered adipo-dermal graft harvested from the supra-pubic area. (**C**) Six months after additional autologous fat grafting to the right temporal region and subsequent weight loss. (**D**) Twelve months after a second triple-layered adipo-dermal graft harvested from the supra-pubic area and autologous fat grafting.

**Figure 4:**
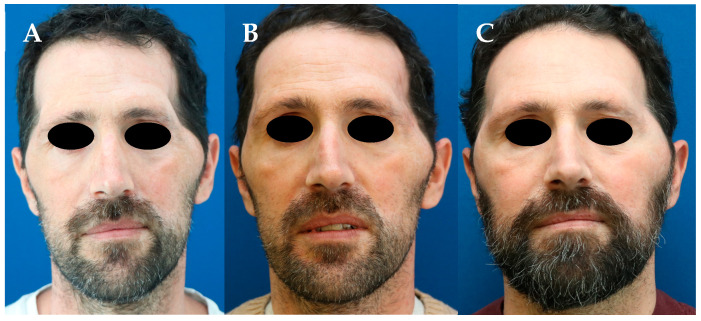
(**A**) Severe bilateral temporal hollowing with peri-zygomatic bulging 1 year after a bilateral fronto-temporal craniectomy. (**B**) Three months after the inset of a bilateral double-layered adipo-dermal graft harvested from the supra-pubic area. (**C**) Twelve months after bilateral autologous fat grafting harvested from the peri-umbilical region.

**Table 1:** Summary of patients’ characteristics, surgical history and outcome.

N° of Patient	Gender	Patient Age (Years)	BMI Pre-op (kg/m^2^)	BMI Post-op (kg/m^2^)	Surgical Approach (Unilateral/ Bilateral)	Donor Site	Graft Layers	N° of Adipo-Dermal Graft Procedures	N° of Autologous Fat Grafting Procedures and Volume (mL)	Follow-Up (Months)	Post-Operative Satisfaction *
1	Male	43	28	31	Unilateral	Abdomen	2 per procedure	2	0	21	2
2	Female	64	23	22	Unilateral	Abdomen	3 per procedure	2	1/30	13	3
3	Male	23	22	23	Unilateral	Abdomen	2	1	2/20; 30	21	3
4	Male	44	25	28	Bilateral	Abdomen	2 each side	1	2/40 bilateral; 30 left, 20 right	27	2
5	Male	34	25	29	Bilateral	Abdomen	2 each side	1	1/60 left, 30 right	24	3

* Postoperative satisfaction: 1 = poor; 2 = sufficient; 3 = good; 4 = excellent.

## Data Availability

Data are contained within the article or Supplementary Materials.

## Supplementary Materials

The following supporting information can be downloaded at: https://www.mdpi.com/article/10.3390/jcm13164936/s1.
