# Supplementary material for: Correction of Post-Surgical Temporal Hollowing with Adipo-Dermal Grafts: A Case Series
Source: J Clin Med. 2024 Aug 21;13(16):4936. doi: 10.3390/jcm13164936 (PMC11355334; doi:10.3390/jcm13164936)
Supplement: Supplementary file 1 [file jcm-13-04936-s001.zip › jcm-3119675-supplementary.pptx]

## Slide 1
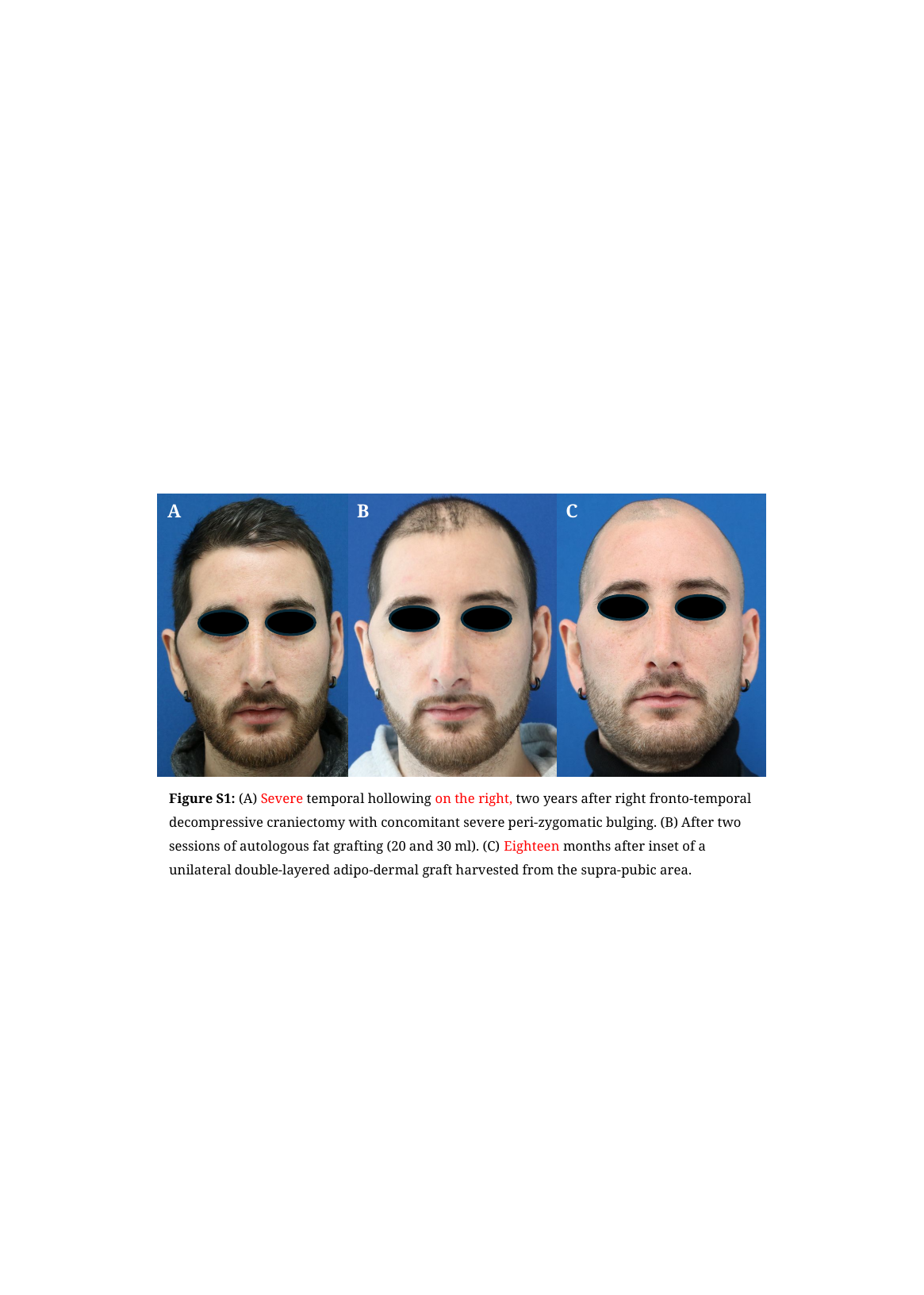

B
A
C
Figure S1: (A) Severe temporal hollowing on the right, two years after right fronto-temporal decompressive craniectomy with concomitant severe peri-zygomatic bulging. (B) After two sessions of autologous fat grafting (20 and 30 ml). (C) Eighteen months after inset of a unilateral double-layered adipo-dermal graft harvested from the supra-pubic area.

## Slide 2
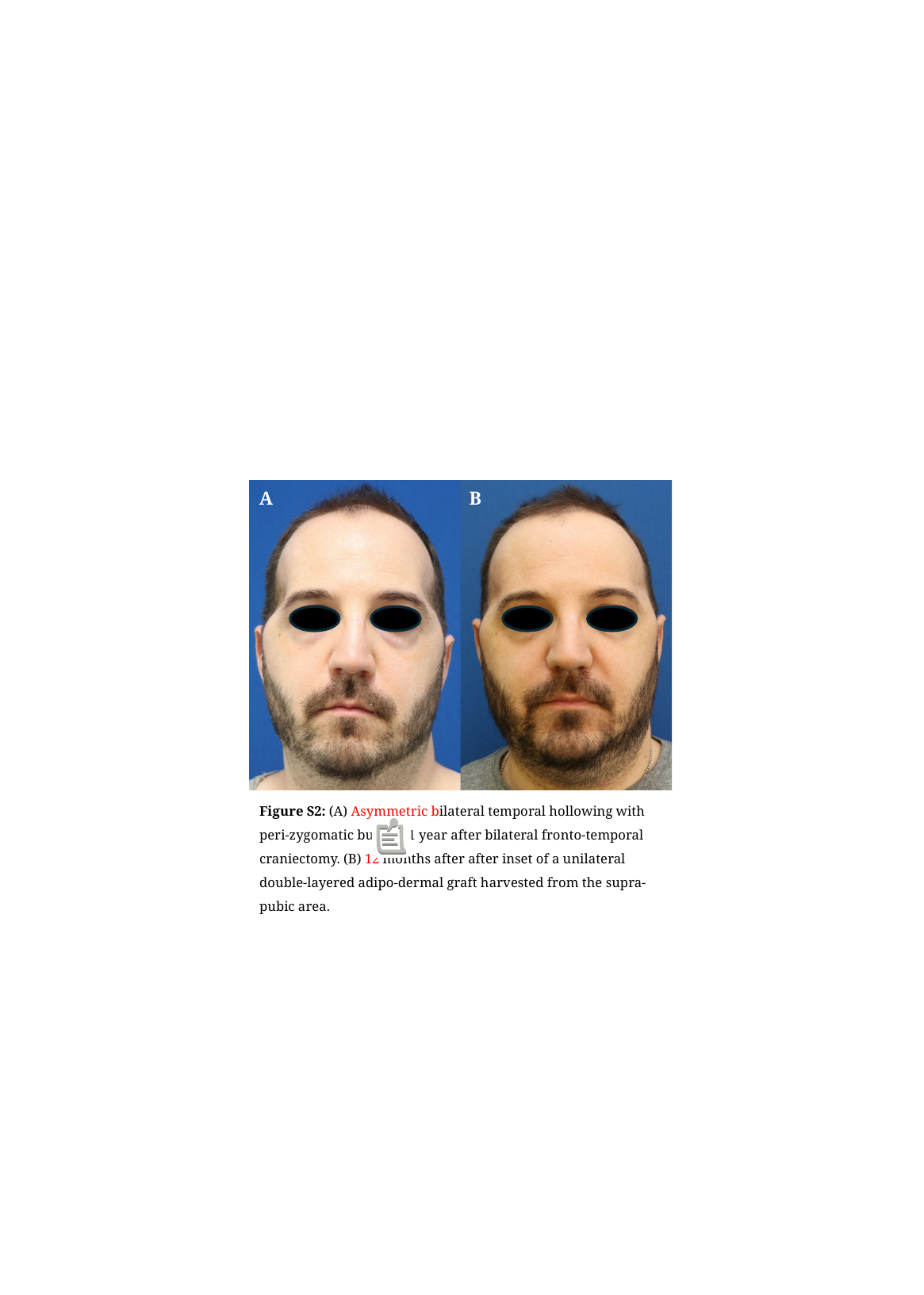

A
B
B
Figure S2: (A) Asymmetric bilateral temporal hollowing with peri-zygomatic bulging 1 year after bilateral fronto-temporal craniectomy. (B) 12 months after after inset of a unilateral double-layered adipo-dermal graft harvested from the supra-pubic area.
